# PERSONS AGING WITH A PHYSICAL DISABILITY: EVALUATING PARTICIPATION CHANGE USING A TOOL FOR COMMUNITY CARE PROVIDERS

**DOI:** 10.1093/geroni/igad104.2899

**Published:** 2023-12-21

**Authors:** Michelle Putnam, Kerri Morgan, Holly Hollingsworth, Rachel Heeb, Szu-Wei Chen, Susan L Stark

**Affiliations:** Simmons University, Boston, Massachusetts, United States; Washington University in St. Louis School of Medicine, St. Louis, Missouri, United States; Washington University in St. Louis School of Medicine, St. Louis, Missouri, United States; Washington University in St. Louis School of Medicine, St. Louis, Missouri, United States; Washington University in St. Louis School of Medicine, St. Louis, Missouri, United States; Washington University in St. Louis School of Medicine, St. Louis, Missouri, United States

## Abstract

Community participation measures for persons aging with disability were developed and evaluated to support community-based organizations (CBOs) with efficient assessment of change in participation and need for supports/services to facilitate participation. This study aimed to evaluate a set of 9 activity domain measures to broadly assess community participation and change in participation over time. A community-based sample (N=323) of persons ages 45-65 responded to a survey with repeated measures three times annually (T1, T2, T3) between 2019-2022. Nine activity domain measures were developed based on extant research and evaluated with assistance from community-based support service providers. Statistical analyses employed T-tests and Chi-square tests to assess change in participation over time, perceptions of participation satisfaction, and assistance needed to facilitate participation. Participants were asked if they thought changes were attributable to aging, the COVID pandemic, or other factors. Findings showed varying levels of participation across the 9 activity domains, with the lowest participation rate for employment and the highest participation rates for personal leisure and managing medications across T1, T2, and T3. Change in participation over the 3-year period was limited; most change was reported as activity reduction. In general, respondents indicated that reduction was due to their aging or the COVID-19 pandemic. Personal assistance, transportation, environmental modifications, and improved health were identified as factors needed to help increase participation levels. Conclusions were that activity domain measures demonstrated efficiency in identifying participation rates and change. CBOs may deem them useful for assessing support and service needs to facilitate participation.

